# Visceral Adiposity Index as an Indicator for Menstrual Disturbance, Hormonal and Metabolic Dysfunction in Polycystic Ovarian Syndrome

**DOI:** 10.7759/cureus.29796

**Published:** 2022-09-30

**Authors:** Priyanka Sharma, Avir Sarkar, Harnam Kaur, Usha Gupta, Binay Kumar

**Affiliations:** 1 Obstetrics and Gynecology, ESIC Medical College and Hospital, Faridabad, IND; 2 Biochemistry, ESIC Medical College and Hospital, Faridabad, IND; 3 Obstetrics and Gynecology, Lady Hardinge Medical College, New Delhi, IND; 4 Cardiology, ESIC Medical College and Hospital, Faridabad, IND

**Keywords:** insulin resistance, free androgen index, metabolic syndrome, visceral adiposity index, polycystic ovarian syndrome

## Abstract

Background: Increase in visceral adiposity is characteristic of polycystic ovarian syndrome (PCOS) and is the main cause of insulin resistance and hyperandrogenism. This study tried to compare the visceral adiposity index (VAI) in PCOS women and control population, thereby exploring its correlation with ovarian morphology, hormonal and metabolic dysfunction.

Materials and Methods: Reproductive-age women who fulfilled the Rotterdam criteria for PCOS constituted the cases. Control population consisted of the same number of non-PCOS women. History of menstrual irregularity and features of hyperandrogenism were noted. Overnight fasting serum hormonal profile on second day of the cycle, oral glucose tolerance test (OGTT) and serum fasting insulin and lipid profile were obtained. Ultrasound evaluation was done simultaneously. Free androgen index (FAI), homeostatic model assessment of insulin resistance (HOMA-IR) and VAI were calculated.

Results: Serum androgen levels and OGTT were greater in PCOS women. No significant difference was noted in serum fasting glucose, fasting insulin and lipid profile between cases and controls. Both systolic and diastolic blood pressures were significantly higher among women with PCOS. Mean ovarian volume, antral follicle count, FAI and HOMA-IR were higher in PCOS women. VAI was significantly higher in cases compared to controls. VAI demonstrated a strong negative correlation with number of menstrual cycles per year. Increasing VAI was associated with longer menstrual cycles and correlated positively with greater severity of anovulation. VAI also showed highly significant correlation with fasting blood glucose and statistically significant moderately strong positive correlation with OGTT values at two hours post glucose challenge, systolic blood pressure and mean ovarian volume. There was no demonstrable correlation between androgen levels or HOMA-IR values.

Conclusion: VAI is higher in women with PCOS. It correlates positively with features of disease severity and ovarian morphology. An assessment of VAI in PCOS women could be predictive of a greater propensity for development of classical metabolic risk factors.

## Introduction

Polycystic ovarian syndrome (PCOS) affects 15-20% of reproductive-age women and has profound and far-reaching metabolic implications [1-3]. Insulin resistance and hyperinsulinemia are key factors in the pathogenesis of PCOS. PCOS has a characteristic increase in visceral adiposity which plays a major role in insulin resistance, compensatory systemic hyperinsulinism and hyperandrogenism [4,5]. Visceral adiposity is a surrogate marker for adipocyte dysfunction in the body. Visceral adiposity is best assessed by visceral computed tomographic scan which is both labor and cost intensive and is therefore not feasible out of a research context. Visceral adiposity index (VAI) is a mathematical model which uses simple anthropometric measurements and serum lipid parameters to reflect the visceral adiposity and insulin resistance. Because of its higher specificity and sensitivity as compared to the classical parameters (like body mass index, waist circumference, and lipids concentration), it is highly useful for cardiometabolic risk assessment [6,7]. VAI is now regarded as a surrogate marker of the function of adipose tissue and is directly proportional to the cardiometabolic risk among reproductive-age women [7]. Visceral adiposity and insulin sensitivity are both considered markers of metabolic risks in PCOS. There is no fixed cut-off value of VAI for the prediction of different parameters of cardiometabolic risk. It is a sex-specific index calculated in females by the formula [8]:

VAI = 36.58(WC) + 1.89(BMI) * 0.81(TG) * 1.52(HDL)

where the WC is waist circumference in centimeters, BMI is basal metabolic index in kilograms/square meters, and TG (triglyceride) and HDL (high-density lipoprotein) concentrations are expressed in mmol/L.

In females with PCOS, visceral adiposity causes premature atherosclerosis, thereby leading to low-grade chronic inflammation in the major blood vessels and increased cardiovascular mortality and morbidity. The metabolism of cortisol and sex steroids in visceral fat is altered. As a result, there is alteration in the secretion of adipokines such as leptin, which directly exert their effects on the ovary [7]. The Androgen Excess and PCOS Society has recommended that assessment of women with high BMI and cardiometabolic risk is mandatory in clinical practice [9]. Therefore, it is recommended now that visceral adiposity assessment should be performed irrespective of overall obesity for early prediction of cardiovascular morbidity in patients with PCOS and plan therapeutic plans accordingly.

## Materials and methods

The study compared the VAI in PCOS women versus non-PCOS controls and explored the correlation of VAI with menstrual disturbance, ovarian morphology, hormonal and cardiometabolic dysfunction in PCOS and non-PCOS women. Systolic and diastolic blood pressures, serum lipid profile and homeostatic model assessment of insulin resistance (HOMA-IR) were used to assess the cardiometabolic dysfunction in PCOS. This case control study was conducted in the Gynecology outpatient clinic of a tertiary care medical college over a period of one year. During the study time frame, 45 reproductive-age women (18-45 years) who fulfilled the 2003 Rotterdam Criteria for PCOS were recruited into the study [10]. The control population consisted of the same number of reproductive-age women (18-45 years) attending the Gynecology outpatient clinic who did not fulfill the 2003 Rotterdam Criteria. Informed consent was obtained from all participants. Institutional ethics committee approval was taken prior to commencement of the study. PCOS was defined as the presence of at least two of the following [10]: oligo or anovulation (less than nine menses per year); hyperandrogenism (hirsutism defined as Ferriman- Gallaway score more than eight with or without acne); hyperandrogenemia (serum testosterone > 0.8 nanogram/milliliter); and polycystic ovaries (>12 follicles of 2 to 9 mm in one or both ovaries or increase in the ovarian volume > 10 milliliters).

Participants were excluded from the study if they had existing endocrine disorders like Type 2 diabetes mellitus, abnormal thyroid function, Cushing’s syndrome, adult-onset congenital adrenal hyperplasia or hyperprolactinemia, any autoimmune disorders, overt hypo or hyperthyroidism, hepatic or renal dysfunction. Participants already on hormonal treatment and/or insulin-sensitizing drugs were also excluded from the study. Clinical data included history of menstrual irregularity, acne or hirsutism, number of menstrual cycles per year, relevant past history and family history, blood pressure (BP), Ferriman Gallaway (FG) Score, presence or absence of acne and acanthosis nigricans. Amenorrhoea was defined as absence of spontaneous menses for six months. Oligomenorrhoea was classified as severe for fewer than six cycles per year and mild for six to eight cycles per year. Hirsutism was defined as FG Score more than eight. Anthropometric data were obtained. BMI was calculated as weight (kilogram)/ square of height (meter).

Laboratory evaluation included overnight fasting serum thyroid stimulating hormone (TSH), luteinizing hormone (LH), follicle stimulating hormone (FSH), prolactin, total testosterone, dehydro-epiandrosterone (DHEAS), androstenedione and sex hormone binding globulin (SHBG) on second or third day of the menstrual cycle. LH over FSH ratio was noted. A 75-gram oral glucose tolerance test (OGTT) and serum fasting insulin and fasting lipid profile were also obtained. Free androgen index (FAI) was calculated. HOMA-IR was calculated using the formula [11]:

HOMA-IR = (Fasting Blood Glusose (mg/dl) * Fasting Serum Insulin (mIU/L))/405

A trans-vaginal (in ever-married women) or a trans-abdominal sonogram (in unmarried women or women unwilling for transvaginal scan) was conducted on the second to fifth day of spontaneous or progesterone induced menses to look for polycystic ovaries and ovarian volume on the Philips Clear Vue 350 scan machine (Philips Ultrasound, Inc, Bothell, WA, USA). Antral follicle count (AFC) included all follicles between 2 to 9 millimeters. If a dominant follicle >10 millimeters or a persisting corpus luteal cyst was seen, the scan was repeated in the next cycle. Statistical analysis was performed by SPSS Package 16 (SPSS Inc., Chicago, IL, USA). Continuous variables were presented as mean ± SD. Rates and proportions were calculated for categorical data. Student T test was used to analyze continuous variables and χ2 Test for categorical variables. Regression analysis was performed to test the association between the variables in PCOS and VAI. P<0.05 was considered statistically significant.

## Results

**Figure 1:**
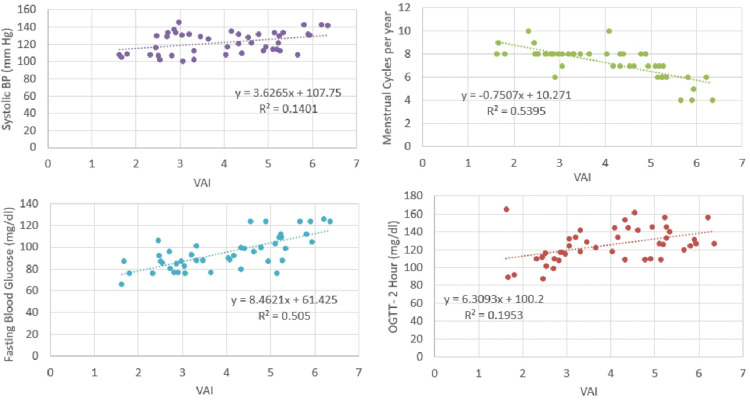
Correlation of VAI with severity of ovulatory and metabolic dysfunction in PCOS women VAI: visceral adiposity index, PCOS: polycystic ovarian syndrome, OGTT: oral glucose tolerance test

The baseline clinical, biochemical and sonographic features of women in both study groups are listed in Table 1. Mean age was similar in both groups. PCOS women had significantly fewer cycles per year and significantly higher BMI than controls. Significantly greater number of PCOS women had hirsutism or acne compared to controls. Serum androgen levels were greater for these women than controls. The two-hour OGTT values were significantly higher in PCOS women. No significant difference was noted in serum fasting glucose, fasting insulin and lipid profile between cases and controls. Both systolic and diastolic BP were significantly higher among women with PCOS. Mean ovarian volume, AFC, FAI and HOMA-IR were higher in PCOS women. VAI was significantly higher in cases compared to controls (Table 1, Figure 1).

**Table 1:** Comparison of clinical, biochemical, sonographic features and VAI in PCOS cases and non-PCOS controls VAI: visceral adiposity index, PCOS: polycystic ovarian syndrome, SHBG: sex hormone binding globulin, DHEAS: dehydro-epiandrosterone, OGTT: oral glucose tolerance test, HDL: high-density lipoprotein, TG: triglyceride, AFC: antral follicle count, HOMA-IR: homeostatic model assessment of insulin resistance, FAI: free androgen index

Parameter	PCOS women (N=45)	Non PCOS Controls (N=45)	p value
Age (years)	24.11 + 4.6	25.24 + 0.25	0.25
Menstrual cycles per year	7.28+1.35	11.64+1.19	<0.001(HS)
Clinical Hyperandrogenism	15	11	0.352
Testosterone (nmol/L)	1.99 + 1.56	0.59 + 0.37	0.08
Androstenedione (nmol/L)	2.38 + 0.72	1.46 + 0.59	<0.001(HS)
SHBG (nmol/l)	44.10 + 17.54	40.78 + 15.07	<0.001(HS)
DHEAS (ng/dl)	172.95 + 29.27	156.00 + 38.76	0.02(S)
Fasting blood glucose (mg/dl)	95.04 + 15.83	89.28 + 15.02	0.08
OGTT- 2 hour (mg/dl)	125.26 + 18.98	117.08 + 14.29	0.02(S)
Fasting insulin (mIU/L)	8.44 + 3.87	7.34 + 2.40	0.11
HDL (mmol/L)	1.06+0.11	1.10+ 0.09	0.08
TG (mmol/L)	2.45+ 0.67	2.33+ 0.60	0.38
Total Cholesterol (mg/dl)	151.57 + 15.71	147.02 + 14.29	0.15
Mean ovarian volume (ml)	15.31 + 4.20	7.50 + 1.79	<0.001(HS)
AFC	16.46 + 6.30	6.13 + 1.91	<0.001(HS)
BMI (kg/m^2^)	25.92 + 2.92	22.90 + 2.87	<0.001(HS)
Systolic BP (mm Hg)	122.15 + 12.88	116.35 + 10.35	0.02(S)
Diastolic BP (mm Hg)	74.42 + 11.91	67.57 + 7.59	0.001(HS)
HOMA- IR	1.97 + 1.01	1.61 + 0.57	0.04(S)
FAI (%)	5.31 + 4.13	1.56 + 1.01	<0.001(HS)
VAI	3.97 + 11.32	3.38 + 1.01	0.02(S)

Among PCOS women, VAI demonstrated a strong negative correlation with menstrual cycles per year. Increasing VAI was associated with longer menstrual cycles, and therefore correlated positively with greater severity of anovulation. VAI also showed highly significant correlation with fasting blood sugars and statistically significant moderately strong positive correlations with two-hour OGTT values, systolic BP, mean ovarian volume and AFC. There was no demonstrable correlation between androgen levels or HOMA-IR values (Table 2). In non-PCOS controls, a weak positive correlation was noted with increasing BMI. VAI also showed a moderate strength positive correlation with OGTT values. No other significant correlations were observed in non-PCOS women (Table 2).

**Table 2:** Correlation of VAI with clinical, biochemical and sonographic features in PCOS women VAI: visceral adiposity index, PCOS: polycystic ovarian syndrome, LH: luteinizing hormone, FSH: follicle stimulating hormone, SHBG: sex hormone binding globulin, DHEAS: dehydro-epiandrosterone, OGTT: oral glucose tolerance test, AFC: antral follicle count, HOMA-IR: homeostatic model assessment of insulin resistance, FAI: free androgen index

	PCOS Women (N=45)		Non PCOS Women (N=45)	
Parameter	r value	p value	r value	p value
Menstrual cycles per year	-0.735	<0.001 (HS)	0.225	0.137
BMI (kg/m2)	0.140	0.359	0.307	0.041(S)
LH/FSH	-0.123	0.301	0.104	0.496
Total testosterone	-0.140	0.358	-0.140	0.360
Androstenedione	0.126	0.410	0.041	0.788
SHBG	0.043	0.781	-0.090	0.555
DHEAS	-0.043	0.777	-0.024	0.878
Fasting blood glucose	0.711	<0.001 (HS)	0.308	0.039(S)
OGTT- 2 hour	0.442	0.002 (S)	0.481	0.001(HS)
Fasting insulin	0.006	0.968	0.007	0.963
Total CH	0.169	0.267	0.193	0.204
Mean Ovarian volume	0.481	0.001 (HS)	-0.259	0.086
AFC	0.518	<0.001 (HS)	-0.069	0.653
Systolic BP	0.374	0.001 (HS)	-0.046	0.763
Diastolic BP	0.137	0.370	-0.160	0.292
HOMA- IR	0.230	0.129	0.119	0.437
FAI	-0.139	0.363	-0.052	0.733

## Discussion

Early onset adiposity has been implicated in the pathogenesis of PCOS and can be predictive of a more severe phenotype. It is also linked to the development of low-grade inflammation, greater cardiometabolic dysfunction and future risk of atherosclerosis [12-15]. Our data shows that as an indicator of visceral fat function, VAI correlates well with severity of menstrual dysfunction and ovarian dysmorphology, and seems to be predictive of impaired glucose tolerance, thus indicating propensity for development of diabetes in the future. More severe phenotypes (O+H+P) have been associated with higher VAI in PCOS women [8,16].

Recent studies have also shown that visceral adiposity is linked to the severity of anovulation, insulin resistance and cardiometabolic risk in women with PCOS [12,16,17]. We noted that increasing VAI was associated with longer menstrual cycles. Amato et al. have reported that oligomenorrhoeic subgroup of PCOS women had higher VAI than other PCOS women [16]. Similarly, another study has noted a significant inverse relation between VAI and luteal phase progesterone levels [8]. It was noted that both fasting and post-prandial blood sugars correlated positively with VAI in PCOS women, a similar trend was seen with increasing VAI in non-PCOS women as well, albeit with weaker strength. An Asian study has similarly also observed a positive correlation between VAI and OGTT correlation [18]. Other authors have demonstrated that VAI correlates inversely with insulin sensitivity evaluated by the euglycemic hyperinsulinemic clamp test [10]. Moreover, VAI cut-off of 1.675 has been proposed for identifying insulin resistance by Matsuda (< 25th percentile) [16]. A significant positive association has also been seen of VAI score with HOMA-IR score (OR: 0.82; 95% CI: 0.71-0.94) [8] and fasting hyperinsulinemia. Our study has not detected such an association. This could be attributed to our smaller sample size. We noted a positive correlation of VAI with systolic BP in PCOS women. Fonseka et al. have also noted this correlation [18]. In our study, increasing VAI correlated significantly with greater mean ovarian volume and antral follicle count. There is little literature exploring this relationship, and this association needs to be investigated further.

This study had a few limitations. It was a single center study. The sample size was less and randomization was done. There is no exact cut-off value of VAI for the prediction of different parameters of cardiometabolic risk in PCOS women. This study did not assess how menstrual irregularity and ovarian dysmorphology would have been affected in patients with high VAI without PCOS. Although VAI cannot be claimed as a diagnostic tool for ovulatory dysfunction, androgen excess, cardiovascular and cerebrovascular events, it indirectly reflects other non-classical risk factors such as altered production of adipose tissue cytokines, plasma-free fatty acids and increased lipolytic activity. Studies have shown correlation with metabolic and reproductive dysfunction, androgen excess, inflammation and liver function in PCOS women [16,19,20]. For these reasons, in the future, it is necessary to determine the precise cut-off reference value for identifying a more severe phenotype with increased cardio-metabolic risk in patients with PCOS. This could be useful for early preventive and therapeutic interventions in this high-risk group.

## Conclusions

VAI, a marker for visceral adipocyte dysfunction, is higher in PCOS women that controls. It correlates positively with features of disease severity such as menstrual irregularity and ovarian dysmorphology. Additionally, it correlates with cardiovascular risk factors such as high blood pressure and higher blood glucose values. An independent assessment of VAI in PCOS women may help to isolate more severe phenotypes and could be predictive of a greater propensity for development of classical cardiometabolic risk factors.
